# 24-Hour Glycemic Variations in Drug-Naïve Patients with Type 2 Diabetes: A Continuous Glucose Monitoring (CGM)-Based Study

**DOI:** 10.1371/journal.pone.0071102

**Published:** 2013-07-30

**Authors:** Kiyotaka Ando, Rimei Nishimura, Daisuke Tsujino, Chiaki Seo, Kazunori Utsunomiya

**Affiliations:** 1 Division of Diabetes, Metabolism, and Endocrinology, Department of Internal Medicine, Jikei University School of Medicine, Tokyo, Japan; 2 Graduate School of Public Health, University of Pittsburgh, Pittsburgh, Pennsylvania, United States of America; University of Michigan Medical School, United States of America

## Abstract

**Objective:**

To investigate a 24-hour glycemic variation in drug-naïve, type 2 diabetic patients by using CGM.

**Methods:**

A total of 30 inpatients with type 2 diabetes were included in the study to analyze the 24-hour CGM data.

**Results:**

The patients’ median age was 58 years old (interquartile range: 42–66 years), and their median HbA1c value was 7.6 (6.7–8.8)%. The median time to postprandial peak glucose levels(Peak Time) for each meal was 70–85 minutes, with the range of postprandial glucose increases(Increase Range) for each meal being 83–109 mg/dL. There was a significant positive correlation between the HbA1c values and Increases Range, Peak Time observed after breakfast and dinner, respectively. When the patients were stratified by a median HbA1c value of 7.6% into 2 groups, Increases Range and Peak Time, after breakfast, were shown to be significantly higher in the high-HbA1c group (H) than in the low-HbA1c (L) group. When the subjects were divided into four groups according to HbA1c levels:1 (<7.0%, n = 8), 2 (7.0–7.9%, n = 8), 3 (8.0–8.9%, n = 8), and 4 (≥9%, n = 6), the average glucose level, pre-meal glucose level and postprandial peak glucose level increased steadily from group 1 to 4 in a stepwise manner.

**Conclusions:**

In drug-naïve, Japanese type 2 diabetic patients, the Peak Time and the Increase Range were maximal after dinner. It was shown that the greater the HbA1c values, the longer Peak time and the higher Increase Range after breakfast and dinner. The average glucose level, pre meal glucose level and postprandial peak glucose level increased steadily as HbA1c level increased.

## Introduction

In recent years, improving postprandial hyperglycemia has been shown to play an important role in preventing cardiovascular events [1]–[4]. One of the angiopathic mechanisms due to postprandial hyperglycemia includes contribution of the oxidative stress. Monnier et al reported that oxidative stress showed Mean Amplitude of Glycemic Excursions (MAGE: measure of diabetic instability) and postprandial blood glucose elevation and a strong positive correlation [5]. Facilitating control of such glucose excursions requires an in-depth understanding of postprandial glucose variations.

However, there are very few studies published to date that investigated postprandial glucose variations in type 2 diabetic patients in depth by using CGM [6], [7].

Besides, these studies included a large number of patients who were receiving oral hypoglycemic agents, which could influence the parameters for glycemic variations, such as time to postprandial glucose peaks or the range of postprandial glucose increases, making it rather difficult to evaluate the status of postprandial glycemic variations in type 2 diabetic patients as it is.

Thus, it is important to have an accurate grasp of glycemic variations in type 2 diabetic patients who are not receiving any anti-diabetic drugs. However, to the best of our knowledge, to date, no study has been reported in which this has been addressed. Therefore, our present study aimed to give a full picture of glycemic variations in type 2 diabetic patients.

## Patients and Methods

A total of 30 inpatients with drug-naïve type 2 diabetes were included in this study to analyze the continuous 24-hour CGM data obtained without measurement errors immediately after placement of the CGM sensors. Of all type 2 diabetic patients admitted for glycemic control and monitored by CGM between January 2007 and January 2012, all eligible patients were enrolled in the study. For CGM, CGMS System Gold (Medtronic Inc) was used, with its sensor attached to the abdomen in each subject, who was instructed to refrain from excessive exercises and between-meal eating and to live as much as usual during CGM monitoring.

The total amount of energy to be taken through meals was determined by using 25–30 kcal/kg as a reference, and the patients were given meals that accounted for ① 1,440 kcal/day, ② 1,600 kcal/day and ③ 1,840 kcal/day. In terms of monthly averages, breakfast accounted for ① 435 kcal (carbohydrates, 50.3%; proteins, 14.8%; and lipids, 35.0%), ② 508 kcal (carbohydrates, 51.8%; proteins, 14.5%; and lipids, 33.8%), or ③ 539 kcal (carbohydrates, 48.5%; proteins, 14.5%; and lipids, 37.0%) at 8 am; lunch accounted for ① 491 kcal (carbohydrates, 61.6%; proteins, 19.4%; and lipids, 19.1%), ② 545 kcal (carbohydrates, 63.3%; proteins, 18.0%; and lipids, 18.6%), or ③ 654 kcal (carbohydrates, 60.7%; proteins, 17.6%; and lipids, 21.7%) at noon; and dinner accounted for ① 513 kcal (carbohydrates, 62.2%; proteins, 19.0%; and lipids, 18.9%), ② 548 kcal (carbohydrates, 64.4%; proteins, 18.4%; and lipids, 17.2%), or ③ 647 kcal (carbohydrates, 61.4%; proteins, 17.1%; and lipids, 21.5%) at 6 pm.

All subjects were instructed to restrict their exercise to the level to which it is usually practiced. A majority of patients were found to have taken 30-minute strolls once or twice daily in the premises. Breakfast, lunch and dinner were provided daily at 8∶00, 12∶00, and 18∶00, respectively.

Parameters to be evaluated in CGM included 24-hour mean glucose levels, standard deviations (SD) of 24-hour glucose levels, MAGE, pre-meal glucose level, postprandial peak glucose level, the range of postprandial glucose increases from premeal, and times to glucose peaks after each meal. To minimize the influence of hospital admission, CGM data obtained in the first 24 hours after admission were used to calculate the SD of 24-hour glucose levels and the MAGE for analysis in principle.

The HbA1c values and the range of glucose increases after each meal from premeal, as well as the HbA1c values and the times to glucose peaks after each meal, were examined for correlation. The HbA1c values used for analysis in this study were those obtained at admission.

The patient population in this study was associated with a median HbA1c value of 7.6%, and the patients were stratified by the median HbA1c levels. The glucose profiles between the groups were compared.

Then the subjects were divided into four groups according to HbA1c levels:1 (<7.0%, n = 8), 2 (7.0–7.9%, n = 8), 3 (8.0–8.9%, n = 8), and 4 (≥9%, n = 6) to examine each parameter related to glycemic variations to identify trends, if any, for each HbA1c stratum.

Statistical analyses were performed by using SPSS Ver. 17. Values for all parameters are represented as medians (interquartile range, 25% to 75%) and were compared by using Kruskal-Wallis test and Mann-Whitney test. Spearman’s correlation coefficients were used to examine correlation between any 2 variables.

Written informed consent was obtained from participants of this study. The present study was conducted with the approval of the Ethics Committee of Jikei University School of Medicine, Tokyo, Japan.

## Results

**Figure 1:**
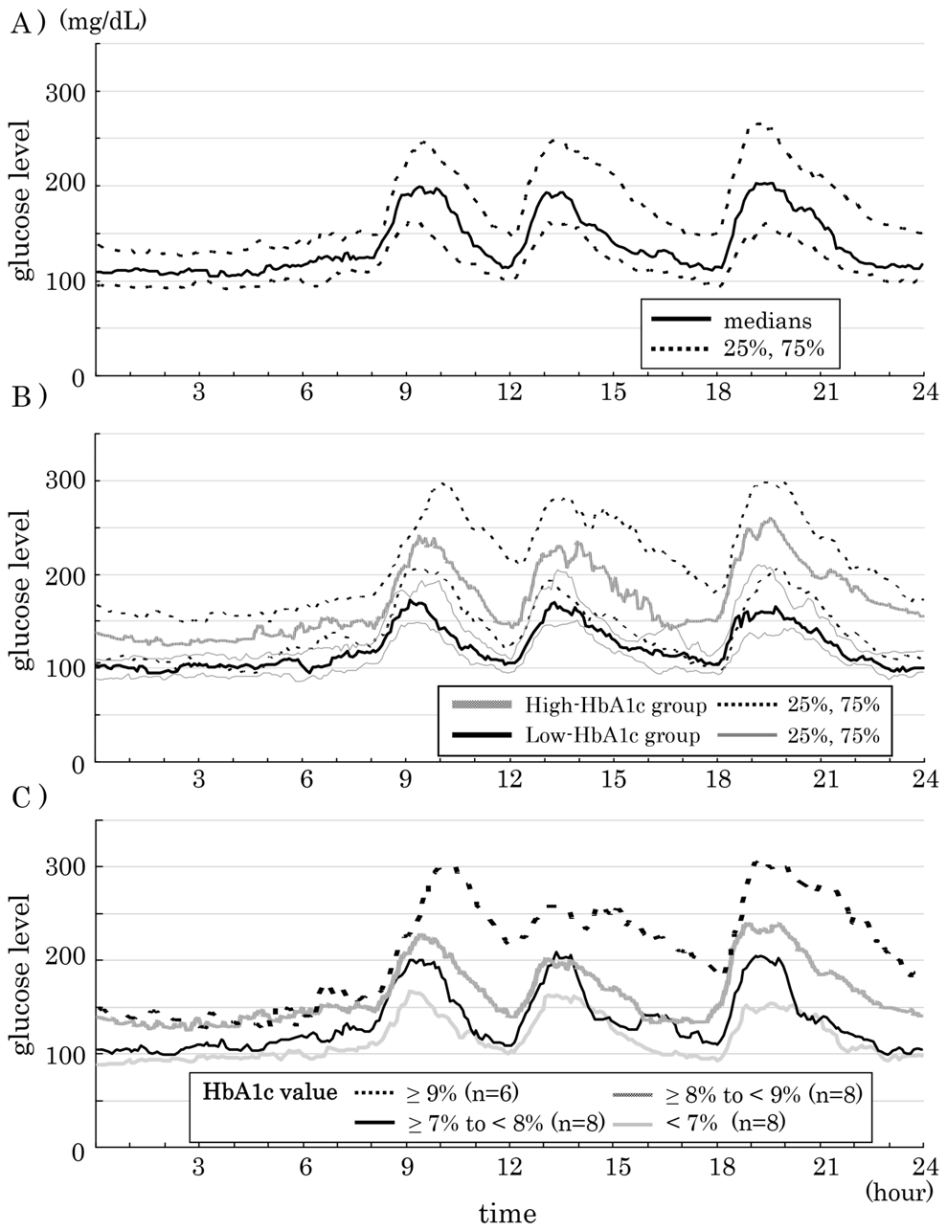
24-hour glycemic variations drug-naïve, type 2 diabetic patients. A) All patients (n = 30). B) Low-HbA1c group (n = 15) and high-HbA1c group (n = 15), Low-HbA1c group: HbA1c 5.7–7.3%, High-HbA1c group: HbA1c 7.8–12.6%. C) Four groups according to HbA1c levels:1 (<7.0%, n = 8), 2 (7.0–7.9%, n = 8), 3 (8.0–8.9%, n = 8), and 4 (≥9%, n = 6).

Of the 30 patients included in the study, 22 were males and 8 were females. Their median age was 58 (interquartile range, 42–66) years; their median BMI, 25.3 (22.6–27.3) kg/m^2^; their median HbA1c, 7.6 (6.7–8.8) %; and their median urinary C peptide immunoreactivity (CPR), 78.6 (60.7–99.8) µg/day. Their median glucose level was 137 (117–179) mg/dL; their SD, 32.5 (26.8–48.5); their median MAGE, 90.8 (75.9–120.3); their highest median glucose level, 218 (188–315) mg/dL; and their lowest median glucose level, 83 (74–119) mg/dL; their median pre-meal glucose level, breakfast 121 (103–149) mg/dL, lunch 109 (96–146) mg/dL, dinner 109 (91–148) mg/dL; their median postprandial peak glucose level, breakfast 205 (172–267), lunch 200 (167–265), dinner 217 (173–280). (Table 1, Figure 1).

**Table 1:** Patient profile and the summary for glycemic variations by the median HbA1c value of 7.6%.

	Overall	Low-HbA1c group	High-HbA1c group	P value*
Patients tested(n)	30	15	15	
Age(years)	58(42–66)	58(50–67)	57(39–65)	0.852
Sex(male/female)	22/8	8/7	14/1	0.011
Duration of diabetes(years)	1.7(0.3–3.3)	1.0(0.3–3.0)	2.0(0.3–6.0)	0.267
BMI(kg/m^2^)	25.3(22.6–27.3)	24.0(21.6–27.9)	25.3(23.5–27.3)	0.477
Urinary CPR(µg/day)	78.6(60.7–99.8)	78.0(44.1–98.7)	79.1(70.2–101.0)	0.363
HbA1c(%)	7.6(6.7–8.8)	6.8(6.1–7.1)	8.8(8.1–9.5)	<0.001
Average glucose level(mg/dL)	137(117–179)	119(112–134)	177(142–233)	0.001
SD(mg/dL)	32.5(26.8–48.5)	30(24–36)	47(30–54)	0.027
MAGE(mg/dL)	90.8(75.9–120.3)	80.3(65.0–94.7)	105.3(90.0–124.7)	0.010
Pre-meal glucose level(mg/dL)				
Breakfast	121(103–149)	112(97–122)	139(119–178)	0.007
Lunch	109(96–146)	100(90–111)	133(104–205)	0.008
Dinner	109(91–148)	103(91–112)	148(99–171)	0.056
Postprandial peak glucose Level(mg/dL)				
Breakfast	205(172–267)	178(149–200)	243(209–317)	0.001
Lunch	200(167–265)	173(156–207)	242(192–318)	0.013
Dinner	217(173–280)	186(154–217)	261(216–314)	0.001
Range of glucose increases from premeal(mg/dL)				
Breakfast	83(59–119)	70(43–94)	98(70–143)	0.017
Lunch	84(66–106)	84(56–103)	84(73–109)	0.590
Dinner	109(84–120)	88(59–111)	115(106–146)	0.005
Time to glucose peaks (minutes)				
Breakfast	83(59–110)	70(50–80)	110(85–125)	0.002
Lunch	70(59–88)	75(60–95)	65(55–75)	0.588
Dinner	85(65–99)	65(55–90)	90(80–120)	0.013

Abbreviations: CPR, C peptide immunoreactivity; MAGE, mean amplitude of glycemic excursions.

Data are shown as medians (interquartile range: 25 to 75 percentiles).

* Mann-Whitney test for comparisons between the low- and high-HbA1c groups; Fisher’s exact test for sex differences.

Low-HbA1c group: HbA1c 5.7–7.3%, High-HbA1c group: HbA1c 7.8–12.6%.

The median range of glucose increases from premeal was 83 (59–119) mg/dL after breakfast, 84 (66–106) mg/dL after lunch, and 109 (84–120) mg/dL after dinner.

The median time to glucose peaks in the patients was 83 (59–110) minutes after breakfast, 70 (59–88) minutes after lunch, and 85 (65–99) minutes after dinner.

The correlation coefficients (*r*) for the HbA1c values and the range of postprandial glucose increases were shown to be 0.421 (*P* = 0.021) after breakfast, 0.020 (*P* = 0.916) after lunch, and 0.456 after dinner (*P* = 0.011), demonstrating a significant positive correlation between the HbA1c values and the range of postprandial increases from premeal after breakfast and dinner.

**Figure 2:**
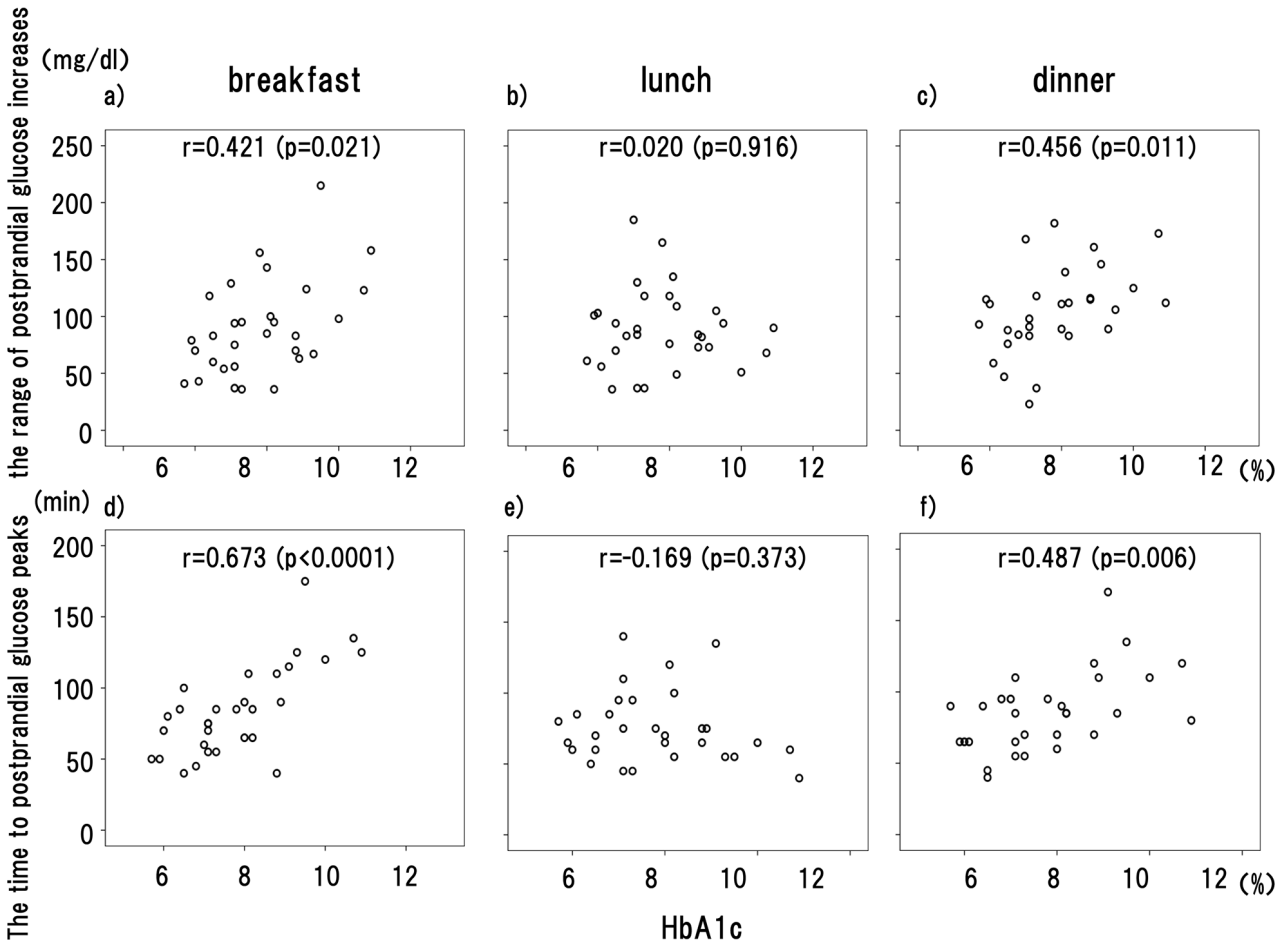
Correlation between the HbA1c and postprandial glucose increases, and time to postprandial glucose peaks. a) Correlation between the HbA1c values and the range of glucose increases after breakfast from premeal. b) Correlation between the HbA1c values and the range of glucose increases after lunch from premeal. c) Correlation between the HbA1c values and the range of glucose increases after dinner from premeal. d) Correlation between the HbA1c values and the time to glucose peaks after breakfast. e) Correlation between the HbA1c values and the time to glucose peaks after lunch. f) Correlation between the HbA1c values and the time to glucose peaks after dinner. Spearman’s correlation coefficients were used to examine correlation between any 2 variables.

The correlation coefficients (*r*) for the HbA1c values and the time to postprandial glucose peaks were shown to be 0.673 (*P*<0.0001) after breakfast, −0.169 (P = 0.373) after lunch, and 0.487 after dinner (*P* = 0.006), demonstrating a significant positive correlation between the HbA1c values and the time to postprandial glucose peaks after breakfast and dinner. (Figure 2).

Then, by using the median HbA1c value 7.6%, the patients were stratified into 2 groups, i.e., those with HbA1c 5.7 to 7.3% (low-HbA1c group) (n = 15) and those with HbA1c 7.8 to 12.6% (high-HbA1c group) (n = 15). The median HbA1c value was 6.8 (6.1–7.1)% in the low-HbA1c group and 8.8 (8.1–9.5)% in the high-HbA1c group.

Again, the median of the average glucose level was 119 (112–134) mg/dL in the low-HbA1c group and 177 (142–233) mg/dL in the high-HbA1c group (*P* = 0.001); their median SD, 30 (24–36) mg/dL in the low-HbA1c group and 47 (30–54) mg/dL (*P* = 0.027) in the high-HbA1c group; their median MAGE, 80.3 (65.0–94.7) in the low-HbA1c group and 105.3 (90.0–124.7) in the high-HbA1c group (*P* = 0.010); their median pre-meal glucose level in the low- and high-HbA1c groups was 112 (97–122) mg/dL and 139 (119–178) mg/dL before breakfast (*P* = 0.007), 100 (90–111) mg/dL and 133 (104–205) mg/dL before lunch (*P* = 0.008), and 103 (89–113) mg/dL and 146 (91–175) mg/dL before dinner (*P* = 0.056), respectively; their median postprandial peak glucose level in the low- and high-HbA1c groups was 178 (149–200) mg/dL and 243 (209–317) mg/dL after breakfast (*P* = 0.001), 173 (156–207) mg/dL and 242 (192–318) mg/dL after lunch (*P* = 0.013), and 186 (154–217) mg/dL and 261 (216–314) mg/dL after dinner (*P* = 0.001), respectively. Thus, all these parameters were shown to be significantly greater in the high-HbA1c group than in the low-HbA1c group. (Table 1, Figure 1).

The median range of postprandial glucose increases in the low- and high-HbA1c groups was shown to be 70 (43–94) mg/dL and 98 (70–143) mg/dL after breakfast (*P* = 0.017), 84 (56–103) mg/dL and 84 (73–109) mg/dL after lunch (*P* = 0.590), and 88 (59–111) mg/dL and 115 (106–146) mg/dL after dinner (*P* = 0.005), respectively, demonstrating that the range of glucose increases from premeal was significantly greater after breakfast and dinner in the high-HbA1c group than in the low-HbA1c group.

The median time to postprandial glucose peaks in the low- and high-HbA1c groups was shown to be 70 (50–80) minutes and 110 (85–125) minutes after breakfast (*P* = 0.002), 75 (60–95) minutes and 65 (55–75) minutes after lunch (*P* = 0.588), and 65 (55–90) minutes and 90 (80–120) minutes after dinner (*P* = 0.013), respectively, showing that the time to postprandial glucose peaks was significantly longer after breakfast and dinner in the high-HbA1c group than in the low-HbA1c group.

When the subjects were divided into four groups according to HbA1c levels, the average glucose level, pre-meal glucose level and postprandial peak glucose level increased steadily from group 1 to 4 in a stepwise manner. (Table 2, Figure 1).

**Table 2:** Patient profile and the summary for glycemic variations according to HbA1c levels:1 (<7.0%), 2 (7.0–7.9%), 3 (8.0–8.9%), and 4 (≥9%).

	1	2	3	4	Pvalue*
HbA1c(%)	<7.0	7.0–7.9	8.0–8.9	≥9.0	
Patients tested(n)	8	8	8	6	
Age(years)	60(35–65)	54(43–66)	60(55–64)	41(39–78)	0.721
Sex(male/female)	5/3	4/4	8/0	5/1	0.115
Duration of diabetes (years)	1.7(0.3–1.2)	2.5(0.3–3.8)	1.7(0.1–3.5)	4.0(1.5–14.5)	0.251
BMI(kg/m^2^)	22.1(20.9–23.8)	27.2(26.3–30.3)	25.0(23.5–26.8)	26.1(22.0–28.4)	0.024
Urinary CPR(µg/day)	51.7(35.7–78.0)	94.4(62.3–115.0)	98.5(77.4–118.0)	70.6(53.1–82.8)	0.013
Average glucose level (mg/dL)	116(109–119)	134(117–162)	166(143–189)	234(128–253)	0.005
SD(mg/dL)	28.5(24.0–32.3)	35.5(21.3–51.8)	31.5(30.0–47.8)	50.0(34.3–69.3)	0.101
MAGE(mg/dL)	75.2(65.5–91.1)	90.5(53.4–128.3)	96.2(85.0–119)	120.7(90.3–134.6)	0.064
Pre-meal glucose level (mg/dL)					
Breakfast	104(90–116)	121(107–143)	144(132–170)	159(109–202)	0.014
Lunch	98(91–101)	109(81–135)	131(109–174)	209(117–254)	0.018
Dinner	91(84–101)	109(103–122)	131(104–158)	181(88–251)	0.044
Postprandial peak glucose level(mg/dL)					
Breakfast	172(143–199)	207(161–259)	231(211–266)	320(195–347)	0.012
Lunch	172(156–192)	207(151–261)	216(191–299)	279(189–345)	0.055
Dinner	176(156–204)	209(157–258)	245(200–294)	323(204–387)	0.022
Range of glucose increases from premeal(mg/dL)					
Breakfast	65(46–82)	85(42–121)	84(65–99)	124(90–172)	0.076
Lunch	77(57–99)	104(49–156)	83(74–116)	82(64–97)	0.518
Dinner	86(63–107)	95(49–156)	114(95–133)	119(102–153)	0.106
Time to glucose peaks (minutes)					
Breakfast	60(46–84)	73(56–83)	88(65–105)	125(119–145)	0.001
Lunch	68(60–84)	85(53–106)	73(65–94)	58(51–83)	0.359
Dinner	65(50–90)	78(58–95)	85(70–105)	115(84–144)	0.056

Abbreviations: CPR, C peptide immunoreactivity; MAGE, mean amplitude of glycemic excursions.

Data are shown as medians (interquartile range: 25 to 75 percentiles).

* Kruskal-Wallis test for comparisons between each groups;

Chi-square test for sex differences.

The study subjects were stratified by sex into two groups for analysis, which, however, demonstrated no difference between the groups with regard to the CGM data.

## Discussion

In this study of drug-naïve type 2 diabetic patients with the median HbA1c 7.6 (6.7–8.8)%, the median of the average glucose level was shown to be 137 (117–179) mg/dL, with the median of their SD being 32.5 (26.8–48.5). In our early study of 24 Japanese subjects with normal glucose tolerance (NGT) [8], the median of the average glucose level was 101 (96–106) mg/dL, with the median of their SD being 16.5 (14.0–19.0). The postprandial glucose peaks were seen in these individuals about 40 to 50 minutes after the start of meals, with the median range of postprandial glucose increases from premeal being 21 (12–32) mg/dL after breakfast, 37 (27–48) mg/dL after lunch, and 44 (25–63) mg/dL after dinner, showing that the range of postprandial glucose increases from premeal was greatest after dinner, followed by that after lunch and smallest after breakfast. Of note, in a study of 434 Chinese subjects with NGT [9], the mean ± SD for the 24-hour glucose levels was shown to be 104±10 mg/dL with the mean SD being 14.2, consistently with the values reported for the Japanese counterparts.

In this study, the range of glucose increases from premeal to glucose peaks was shown to be 83–109 mg/dL in Japanese type 2 diabetic patients, with the time to postprandial glucose peaks being 70–85 minutes, demonstrating that the range of glucose increases was about 2- to 4-fold higher and the time to postprandial glucose peaks was about 20–40 minutes longer in these patients than in the NGT subjects mentioned above [8].

In contrast, the range of glucose increases from premeal was greatest after dinner, followed by that after lunch and smallest after breakfast in these patients, as was the case with the NGT individuals.

In a study of 131 Caucasian type 2 diabetic patients by Monnier et al [6], the peak glucose level was shown to be greatest after breakfast, with the time to postprandial glucose peaks being also longest after breakfast, in contrast to the CGM data in Japanese type 2 diabetic patients presented in this study. This disparity may be accounted for by the fact that the patients in this study were not receiving oral hypoglycemic agents and that the calorie intake varied among the meals taken during the study.

In this study, breakfast was designed to be lower in calorie than lunch or dinner, to obtain data in a setting as close to real life as possible, by taking into account everyday diet habits in Japan that prefer quick, low-calorie meals for breakfast, and slow, high-calorie meals for dinner. It is of note here that the range of glucose increases from premeal was shown to be greatest after dinner in our study of Japanese NGT individuals [8], as in our Japanese type 2 diabetic patients.

In a study examining correlation between postprandial glucose levels, fasting glucose levels and HbA1c values in 290 type 2 diabetic patients [10], Monnier et al reported that the postprandial glucose levels accounted for as much as 70% of the HbA1c values in those with HbA1c less than 7.3% but no more than 40% in those with HbA1c over 9.3%, suggesting that, primarily, restoring the fasting glucose levels to as close to normal as possible leads to improvements in HbA1c values in those with poorly controlled disease (HbA1c, 8% or greater), while suppressing postprandial hyperglycemia leads to improvements in HbA1c values in those with relatively well controlled disease (HbA1c, about 7%) who are less associated with increases in fasting glucose levels.

Again, in a study of 164 type 2 diabetic patients receiving rigorous glycemic control by Woerle et al [11], of the patients who had achieved fasting glucose levels less than 100 mg/dL, only 64% achieved HbA1c levels less than 7%, while, of those who had achieved postprandial glucose levels 140 mg/dL, 94% achieved HbA1c levels less than 7%, suggesting that treatments targeted at postprandial hyperglycemia contribute greatly toward more favorable HbA1c values in type 2 diabetic patients.

In the present study, postprandial glucose peaks were shown to occur in drug-naïve, Japanese type 2 diabetic patients well before 120 minutes after meals, which has represented a major therapeutic target over the years, a finding that may prove helpful in setting postprandial glucose goals that lead to more favorable HbA1c values in type 2 diabetic patients.

The patient population in this study was associated with a median HbA1c value of 7.6% and was stratified by the median HbA1c value into low- and high-HbA1c groups for comparison.

Study results showed that the high-HbA1c group was associated with greater 24-hour glycemic variations than the low-HbA1c group, with the postprandial glucose peaks being delayed after breakfast and dinner in the high-HbA1c group compared to those in the low-HbA1c group. Furthermore, when the subjects were divided into four groups according to HbA1c levels, the postprandial peak glucose level increased steadily from group 1 to 4 in a stepwise manner. These results appear to provide a clear measure of postprandial hyperglycemia based on patient stratification by HbA1c, as well as potential clues as to how to forge a more appropriate approach to postprandial hyperglycemia in type 2 diabetic patients.

Additionally, while there was no significant difference in the parameters examined after lunch between the low- and high-HbA1c groups in this study, this could be due to the fact that the study required that all patients be examined in an inpatient setting to minimize the difference in calorie intake among the patients, which imposed constraints on the scope of their activity times, and many patients were found doing exercises after lunch, but asked to refrain from excessive exercises.

Of note, study results showed that the greater the HbA1c values, the longer the time to glucose peaks, and the greater the range of glucose increases, after breakfast and dinner.

In this regard, Wang et al analyzed 24-hour glycemic variations in 121 type 2 diabetic patients by using CGM and showed that the time to postprandial glucose peaks was 89 to 93 minutes in those with HbA1c less than 8% and 96 to 100 minutes in those with HbA1c 8.1% or greater [7], suggesting that there is a positive correlation between the HbA1c values and the time to postprandial glucose peaks, in agreement with the findings in our study.

The limitation of our study is that it involved a small sample size and inpatients only. However, future studies in a larger population may help to delineate more closely the glycemic variations in type 2 diabetic patients as stratified by HbA1c values.

In summary, data from this study in Japanese type 2 diabetic patients may provide a basis for a variety of clinical studies aimed at evaluation of glycemic variations in a Japanese population as well as clues for appropriate therapeutic intervention in the population.

Further long-term study is required in a similar study population to clarify how the time to postprandial glucose peaks, as well as the range of postprandial glucose increases, in type 2 diabetic patients, may be linked to cardiovascular events in these patients.
